# Optimal duration of prior endocrine therapy predicts the efficacy of Fulvestrant in a real‐world study for patients with hormone receptor‐positive and HER2‐negative advanced breast cancer

**DOI:** 10.1002/cam4.3491

**Published:** 2020-10-06

**Authors:** Yannan Zhao, Yi Li, Chengcheng Gong, Yizhao Xie, Jian Zhang, Leiping Wang, Jun Cao, Zhonghua Tao, Biyun Wang, Xichun Hu

**Affiliations:** ^1^ Department of Medical Oncology Fudan University Shanghai Cancer Center Shanghai China; ^2^ Department of Oncology Shanghai Medical College Fudan University Shanghai China

**Keywords:** fulvestrant, hormone receptor‐positive, metastatic breast cancer, real‐world effectiveness

## Abstract

Fulvestrant 500 mg is standard of care for endocrine therapy‐naive or pretreated women with hormone receptor‐positive (HR+) metastatic breast cancer (MBC). This study was conducted to explore the potential factors and duration of last endocrine therapy as predictors for the efficacy of fulvestrant 500 mg on Chinese patients in real‐world practice. Two hundred and fifty‐two MBC patients who were treated with fulvestrant 500 mg consecutively between January 2011 and December 2015 in our institute were included in this study. Efficacy outcomes included progression‐free survival (PFS), overall survival (OS), and clinical benefit rate (CBR). The optimal cut‐off value for duration of last endocrine therapy was determined by survival ROC analysis. Adverse events were graded according to NCI‐CTC AE 4.0. Fulvestrant 500 mg demonstrated a median PFS of 5.8 months (95%CI 4.6‐6.9), and a median OS of 35.9 months (95%CI 30.2‐41.4). CBR was 41.3% (95%CI 35‐47). Liver metastasis, bone alone metastasis, lines of endocrine therapy for MBC, and sensitivity to last endocrine therapy were statistically significant in the Cox multivariate analysis (P values of 0.022, 0.02, 0.03, and 0.038, respectively). The optimal cut‐off values for duration of last endocrine therapy to predict the efficacy of fulvestrant 500 mg were 25.08 months for adjuvant endocrine therapy and 5.17 months for first‐line endocrine therapy, which showed no difference in prediction power with ABC clinical definition. Patients with prior adjuvant endocrine therapy ≥25.08 months or first‐line therapy≥5.17 months reached a longer PFS of fulvestrant (*p* = 0.04). Six patients discontinued the treatment due to intolerable adverse events. Patients with the duration of prior endocrine therapy longer than optimal cut‐off points indicate better PFS of fulvestrant. Liver metastasis, bone alone metastasis, line of fulvestrant, and sensitivity to last endocrine therapy were also predictors for response of fulvestrant.

ClinicalTrials.gov Identifier: NCT03708432.

## Graphical abstract

This study provided first‐hand data regarding the efficacy and safety profile of fulvestrant 500 mg in Chinese patients with HR+/HER2‐ MBC. We successively collected all patients treated with fulvestrant within a certain period of time to delineate the pattern of real‐world practice. The study identified potential determinants that were significantly associated with PFS of fulvestrant 500 mg.

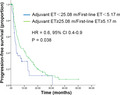

## 1 INTRODUCTION

Hormone receptor‐positive, HER2 (ERBB2)‐negative (HR+HER2−) breast cancer accounts for 70% of all breast cancers.^1^ Endocrine therapy is the mainstay of treatment for HR+HER2− breast cancer.^2^ Though adjuvant endocrine therapy reduces the relative risk of recurrence by approximately 40%, one‐third of the patients finally relapse.^3^ In metastatic setting, endocrine therapy is often successful initially, but inevitably fails due to resistance. Hence, more effective treatment strategies are needed to overcome the resistance to endocrine therapy for breast cancer.

Fulvestrant is an endocrine agent used for the treatment of patients with HR+metastatic breast cancer (MBC). It has a novel mode of action since it binds, blocks, and accelerates degradation of estrogen receptor protein, leading to estrogen signaling inhibition.^4^, ^5^ Fulvestrant demonstrated efficacy in postmenopausal women with endocrine therapy‐naive or pretreated breast cancer.^6^, ^7^

A phase 3 study (FALCON)^6^ compared fulvestrant 500 mg with anastrozole in endocrine therapy‐naive patients. Fulvestrant 500 mg showed a progression‐free survival (PFS) of 16.6 months when compared to the anastrozole group of 13.8 months, and was associated with significant improvement in the PFS (hazard ratio [HR] 0·797, 95% CI 0·637–0·999, *p* = 0·0486). Hence, fulvestrant 500 mg proved to be an effective endocrine agent and remained a standard for the first‐line treatment of HR+patients.

CONFIRM trial^7^ compared fulvestrant 500 mg with fulvestrant 250 mg in postmenopausal women with advanced breast cancer who experienced progression after prior endocrine therapy. Fulvestrant 500 mg significantly prolonged PFS when compared with fulvestrant 250 mg (mPFS 6.5 vs. 5.5 months). Fulvestrant 500 mg also showed a favorable clinical efficacy in postmenopausal MBC patients who progressed on prior endocrine therapy.

Besides, fulvestrant significantly improved OS of patients with HR+HER2− metastatic breast cancer,^8^, ^9^, ^10^ and a subset of patients when given endocrine therapy alone acquired long durable disease control. Further research is warranted to study if there are any patterns for patients to distinguish who will benefit from fulvestrant, or who will be resistant and receive more intensive regimens, such as chemotherapy or combined targeted therapy. And second, duration of disease control for patients who had prior endocrine therapy is an important predictor of endocrine therapy sensitivity. According to the consensus, we always define two years in adjuvant setting and 6 months in metastatic setting as the cut‐off points to separate primary and secondary resistance.^2^ To date, no study has described on optimal cut‐off points of prior endocrine therapy duration, especially the last‐line therapy, to predict the efficacy of fulvestrant. Given that the real world study focuses on the “real effectiveness” in clinical practise, and helps us better understand the potential determinants of treatment outcomes. Therefore, this study aimed to provide additional clinical data of HR+MBC patients treated with fulvestrant 500 mg and explore the potential factors affecting its efficacy in Chinese patients.

## 2 PATIENTS AND METHODS

### 2.1 Patients and treatment

Our analysis comprised HR+HER2− advanced breast cancer patients who were treated with fulvestrant 500 mg from January 2011 to December 2015 in Fudan University Shanghai Cancer Center. All data were retrospectively collected from the medical records. Advanced breast cancer is defined as unresectable, locally advanced breast cancer, de novo stage IV breast cancer, and recurrent BC. HR+ is defined as ER‐positive and/or PR‐positive. Fulvestrant was administered by intramuscular injection in a 500 mg regimen that incorporates a day 14 loading element (500 mg on days 0, 14, and 28, and every 28 days thereafter). All the premenopausal patients received concurrent luteinizing hormone‐releasing hormone analogs (LHRHa).The patients received treatment until disease progression, intolerable toxicity, or voluntary refusal. All patients provided written informed consent before collection and the process of study was approved by the relevant independent ethics committees (No. 1812195‐6).

Assessment variables included PFS, OS (overall survival), ORR (objective response rate), and CBR (clinical benefit rate). PFS is defined as time from first fulvestrant administration to disease progression or death due to various causes, whichever is earlier (Disease progression is according to RECIST 1.1 criteria for tumor assessment). OS is defined as time from first fulvestrant administration to death due to various causes or the last visit of follow‐ups. ORR is defined as the percentage of evaluable patients at baseline who had a best objective tumor response of either complete response (CR) or partial response (PR). CBR is defined as the percentage of evaluable patients at baseline who had a best objective tumor response of complete response (CR), partial response (PR) or stable disease (SD) ≥24 weeks.

### 2.2 Statistical analysis

Descriptive statistics were used to summarize patient characteristics. Kaplan‐Meier plots revealed median PFS and median OS with corresponding 95% confidence intervals and P values for all patients. A multivariate Cox proportional hazard model was developed using stepwise regression (forward selection) to explore independent predictors of PFS. Effects of variables were expressed as hazard ratios with corresponding 95% confidence intervals and P values. Significant variables in the univariate analysis entered into the model. The enter limit and remove limit were *p* = 0.10 and *p* = 0.15, respectively. Potential variables of prognostic significance included: age (<65 years or ≥65 years), menstrual state (postmenopausal vs. premenopausal), liver metastasis (no vs. yes), bone alone metastasis (no vs. yes), sensitivity to last endocrine therapy (no vs. yes), lung metastasis (no vs. yes), lines of endocrine therapy for MBC (=1 vs. ≥2), previous endocrine therapy (TAM/TOR vs. AI/Both), sensitivity to last endocrine therapy prior to fulvestrant (yes vs. no) and previous lines of chemotherapy for MBC (=0 vs. ≥1). Sensitive to endocrine therapy is defined as a relapse ≥12 months of completing adjuvant endocrine therapy, or PD ≥6 months after initiating endocrine therapy for MBC. The overall response rate (ORR) and clinical benefit rate (CBR) were calculated with its 95% CI. SPSS software (SPSS version 21.0, SPSS Inc., Chicago, IL) was used for statistical evaluations.

One of the purposes is to evaluate whether the duration of last endocrine therapy prior to fulvestrant can be a predictor of PFS for fulvestrant. We investigated the optimal cut‐off values and the predictive accuracy of last endocrine therapy duration using survival receiver operating characteristic (ROC) analysis.^11^, ^12^ We used the area under the curve at 6 months to measure predictive accuracy. We used R software version 3.1.3 and the “survival ROC” package to perform survival ROC curve analysis.

## 3 RESULTS

### 3.1 Patients

A total of 252 eligible patients from January 2011 to December 2015 were enrolled in this study at Fudan University Shanghai Cancer Center. Baseline patient characteristics are presented in Table 1. Median age of the patients was 57 years (range 31‐85). Most patients were grade III and stage II‐III. The most common histology was invasive ductal carcinoma. Majority (85.0%) of the patients were postmenopausal. 71.4% (180/252) of patients had at least two metastatic sites. 60.3% (152/252) of the patients had visceral metastasis, which included 43.7% (110/252) of patients with lung metastasis. 72.6% (183/252) patients experienced ≥2 lines of endocrine therapy for MBC before initiation of the current treatment. Most patients (62.7%) were sensitive to previous endocrine therapy according to the definition in this study. 12.7% (32/252) patients received TAM/TOR prior to fulvestrant, 41.7% (105/252) patients received AI, and 39.6% (100/252) patients received two agents above. 55.9% (141/252) of patients had previous chemotherapy for MBC.

**TABLE 1:** Patient characteristics

Characteristic	No. (%)
Median age, years (range)	57 (31‐85)
Age	
<65 y	188 (74.6)
≥65 y	64 (25.4)
Disease‐free interval^a^	
Median (year)	4.2
Range (year)	0.3‐23.1
De novo stage IV	17(6.7)
≤24 mo	49(19.4)
>24 mo	181(71.8)
Not evaluable	5(2.0)
Grade of primary breast tumor	
I	5 (2.0)
II	90 (35.7)
III	153 (60.7)
Unknown	4 (1.6)
Histology of primary breast tumor	
Invasive ductal carcinoma	232 (92.1)
Invasive lobular carcinoma	13 (5.1)
Other types	7 (2.8)
Stage of primary breast tumor	
I	31 (12.3)
II	98 (38.9)
III	102 (40.5)
De novo stage IV	17 (6.7)
Unknown	4 (1.6)
Menstrual state	
Postmenopausal	214 (85.0)
Premenopausal	38 (15.0)
No. of metastatic sites	
1	72(28.6)
2	89(35.3)
≥3	91(36.1)
Metastatic sites	
Visceral	152(60.3)
Lung	110(43.7)
Liver	72(28.6)
Brian	8 (3.2)
Non‐visceral	100(39.7)
Bone alone	42(15.9)
Line of endocrine therapy for MBC	
First‐line	69(27.4)
Second‐line	105(41.7)
Third‐or more‐line	78(31.0)
Sensitivity to last endocrine therapy prior to fulvestrant^b^	
Yes	158 (62.7)
No	71 (28.1)
Not evaluable	23 (9.1)
Previous endocrine therapy^c^	32(12.7)
TAM/TOR	
AI	105(41.7)
Both^d^	100(39.6)
No	15(6.0)
Previous lines of chemotherapy for MBC	
0	111(44.0)
1	62(24.6)
≥2	79(31.3)

Abbreviation: AI, aromatase inhibitor; TAM, tamoxifen; TOR, Toremifene.

^a^ Disease‐free interval is defined as the time from diagnosis of breast cancer to first relapse.

^b^ Sensitivity to previous endocrine therapy was defined as at least 24 mo of endocrine therapy before recurrence in the adjuvant setting or a response or stabilization for at least 24 wks of endocrine therapy for advanced disease.

^c^ Including adjuvant and metastatic setting.

^d^ Both indicated the cases that patients may receive TAM/TOR as adjuvant endocrine therapy and AI as first‐line therapy for MBC.

The data cut‐off time was May 18, 2017. At the cut‐off time, fulvestrant was still ongoing in 17 patients. Treatment was discontinued due to disease progression (71.0%), intolerable toxicity (2.4%), unwillingness to follow the treatment plan (2.0%), economic reasons (9.5%), and loss of follow‐up (8.3%).

### 3.2 Efficacy

**FIGURE 1:**
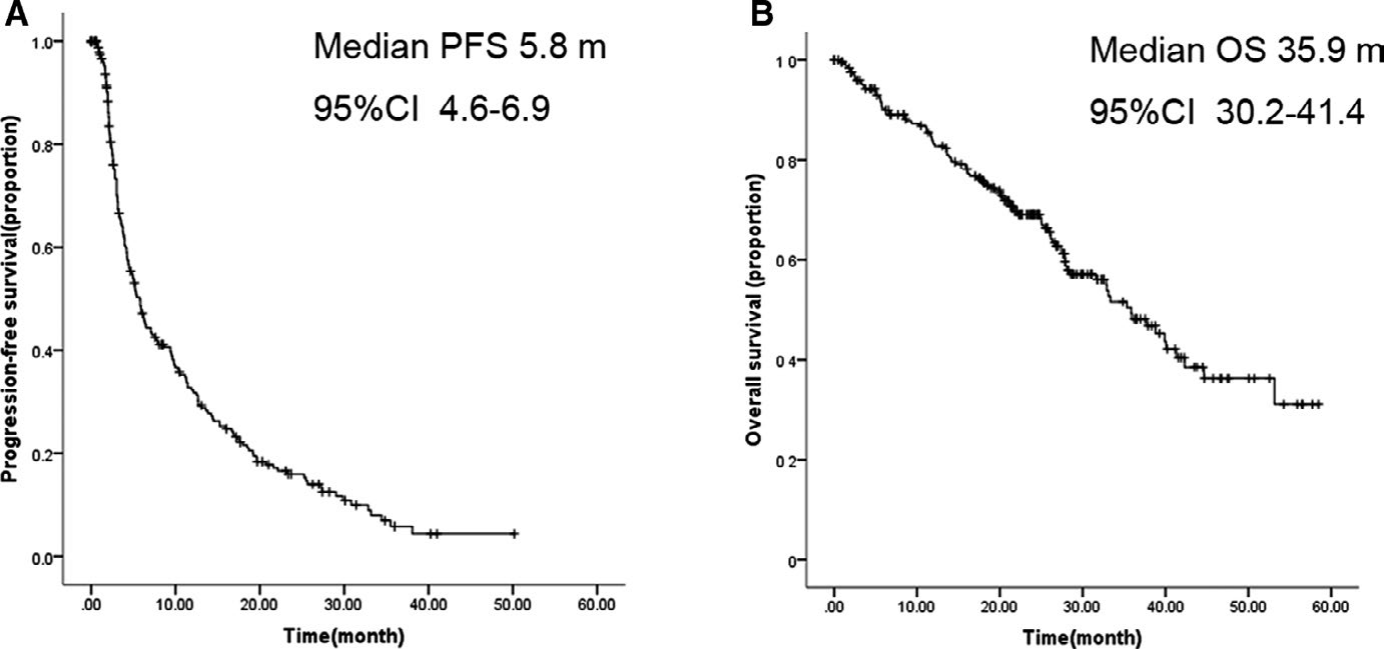
Kaplan‐Meier plot for PFS and OS with fulvestrant 500 mg. A, Kaplan‐Meier plot for PFS; B, Kaplan‐Meier plot for OS. Abbreviations: PFS, progression‐free survival; OS, overall survival

At median follow‐up of 21.7 months, median PFS was 5.8 months (95%CI 4.6‐6.9), and median OS was 35.9 months (95%CI 30.2‐41.4) (Figure 1A,B). CBR was 41.3% (95%CI 35‐47) (Table 2).

**TABLE 2:** Evaluation of efficacy

Variable	No. (%)
Progression‐free survival	
Events—No. (%)	194(77.0)
Duration—mo	
Median	5.8
95%CI	4.6‐6.9
Overall survival	
Events—No. (%)	100(40.0)
Duration—mo	
Median	35.9
95%CI	30.2‐41.4
Best overall response‐No. %	
Complete response	0(0.0)
Partial response	11(4.4)
Stable disease	112(44.4)
Duration of SD ≥24 wks	93(36.9)
Progression disease	93(36.9)
NE	36(14.3)
CBR	104(41.3)

Abbreviations: CBR, clinical benefit rate; CR, complete response; PD, progression disease; PR, partial response; SD, stable disease.

**FIGURE 2:**
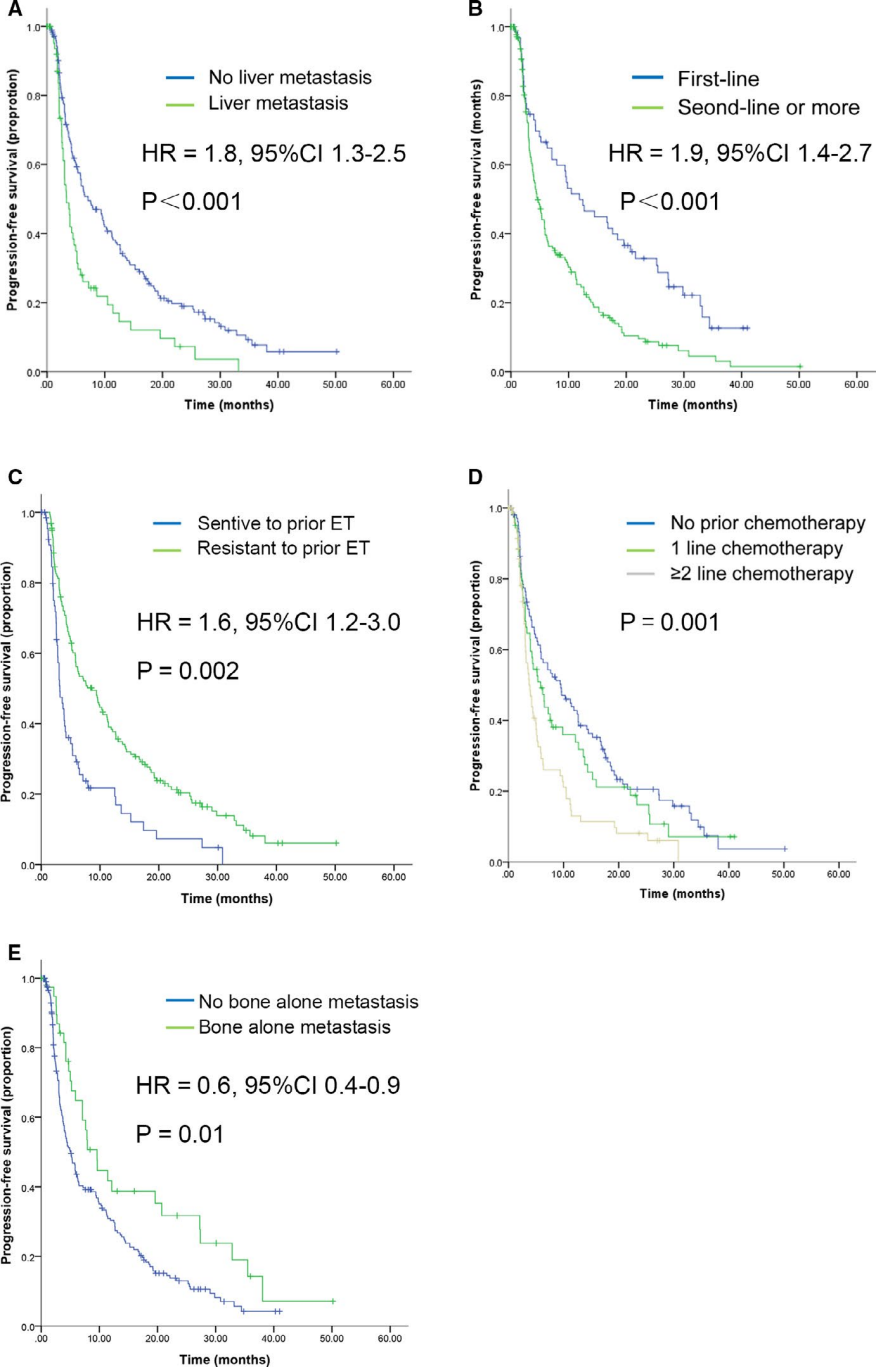
Kaplan–Meier curves for PFS. For patients stratified by potential factors related to PFS. A, Liver metastasis, B, Lines of endocrine therapy, C, Sensitivity to last endocrine therapy, D, Lines of prior chemotherapy, E, Bone alone metastasis. Abbreviations: HR, hazard ratio; CI, confidence interval; PFS, progression‐free survival

Patients with no liver metastasis had better PFS compared to those with liver metastasis (Figure 2A). Median PFS was 3.7 months versus 7.1 months in patients with or without liver metastasis (*p* < 0.001). Patients who received prior endocrine therapy for MBC demonstrated a worse PFS compared to those who did not (Figure 2B). Median PFS was 11.9 months in patients with first‐line endocrine therapy, while 4.7 months in patients with subsequent endocrine therapy (*p < *0.001). Patients sensitive to last endocrine therapy reached a median PFS of months, significantly longer than patients who were not sensitive (median PFS, 7.6 months vs. 3.6 months, *p* = 0.002) (Figure 2C). Median PFS was decreased significantly with successive chemotherapy regimen (Figure 2D). For patients receiving no, first‐, and second‐line chemotherapy or beyond, median PFS was 9.5, 5.8, and 3.8 months, respectively (*p* = 0.001). Bone alone metastasis indicated a longer PFS and median PFS was 9.6 months for patients with it and 5.1 months for those with other metastasis sites (*p* = 0.01) (Figure 2E).

Univariate analysis (Table 3) revealed that no liver metastasis, bone alone metastasis, first‐line fulvestrant administration, previous TAM/TOR use, sensitive to last endocrine therapy and no palliative chemotherapy prior to fulvestrant was associated with significantly longer PFS. Yet, menstrual state and lung metastasis did not influence PFS. Multivariate analysis (Table 3) demonstrated that liver metastasis, bone alone metastasis, lines of endocrine therapy for MBC, sensitivity to last endocrine therapy for MBC were independent predictive factors for PFS.

**TABLE 3:** Univariate and multivariate analysis of factors predicting PFS of fulvestrant 500 mg

	N	Event	Univariate			Multivariate		
			HR	95%CI	*p*	HR	95%CI	*p*
Age								
<65 y	188	148	0.8	0.6‐1.2	0.26			
≥65 y	64	64						
Menstrual state								
Postmenopausal	214	168	0.7	0.4‐1.1	0.15			
Premenopausal	38	26						
Liver metastasis								
No	180	141	1.8	1.3‐2.5	<0.001*	1.5	1.1‐2.1	0.022*
Yes	72	53						
Lung metastasis								
No	142	110	1.0	0.7‐1.3	0.97			
Yes	110	84						
Bone only metastasis								
No	210	165	0.6	0.4‐0.9	0.01*	0.6	0.4‐0.9	0.02*
Yes	42	29						
Line of endocrine therapy for MBC								
1	69	49	1.9	1.4‐2.7	<0.001*	1.5	1.1‐2.5	0.03*
≥2	183	145						
Previous endocrine therapy								
TAM/TOR	32	23	1.4	1.1‐1.7	0.004*	1.6	0.8‐2.9	0.14
AI/Both	205	161						
Sensitivity to last endocrine therapy prior to fulvestrant								
Yes	158	130	1.6	1.2‐3.0	0.002*	1.4	1.0‐2.0	0.038*
No	71	54						
Previous lines of chemotherapy for MBC								
0	111	84	1.4	1.1‐1.6	<0.001*	1.1	0.8‐1.6	0.58
≥1	141	110						

Abbreviations: CI, confidence interval; HR, hazard ratio; MBC, metastatic breast cancer.

* *p* < 0.05 is considered significant.

**FIGURE 3:**
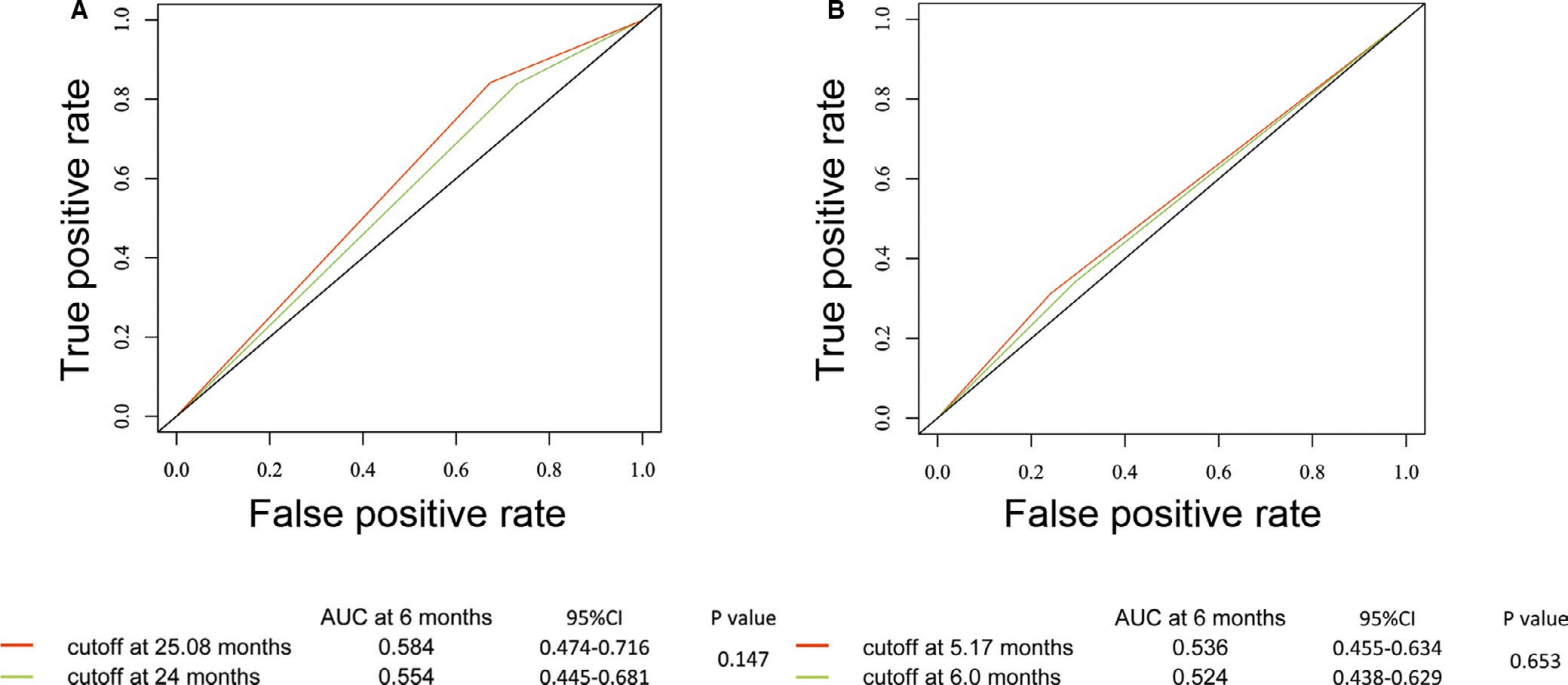
Survival ROC curves compared the prognostic accuracy of two cut‐off points of duration of prior endocrine therapy in patients treated with fulvestrant 500 mg. A, Comparisons of the prognostic accuracy by two cut‐off points of duration of prior adjuvant endocrine therapy (25.08 vs. 24 months) in 56 patients; B, Comparisons of the prognostic accuracy by two cut‐off points of duration of prior adjuvant endocrine therapy (5.17 vs. 6 months) in 103 patients. Abbreviations: ROC, receiver operator characteristic; AUC, area under curve

**FIGURE 4:**
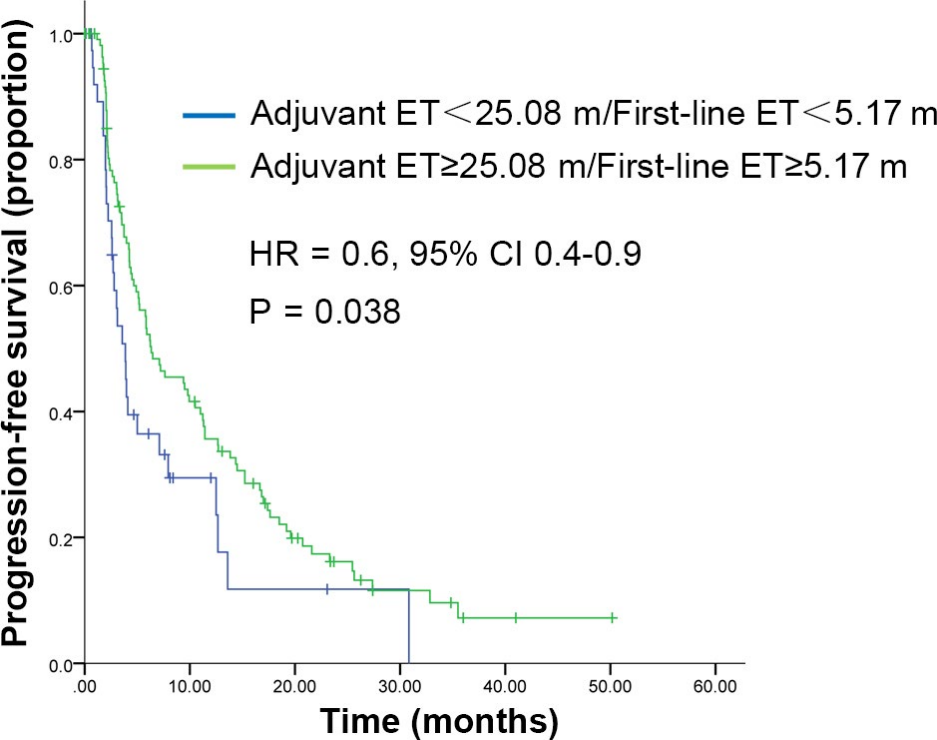
Kaplan–Meier curves for progression‐free survival. For patients stratified by adjuvant ET duration and first‐line ET duration. Abbreviations: HR, hazard ratio; CI, confidence interval; PFS, progression‐free survival.ET, endocrine therapy

In order to investigate the correlation between the duration of last endocrine therapy and PFS, we selected two subgroups: 56 patients who relapsed on adjuvant endocrine therapy and 103 patients who progressed on first‐line endocrine therapy. All these patients had detailed records of prior endocrine therapy. The optimal cut‐off values by survival ROC analysis were 25.08 months for adjuvant endocrine therapy and 5.17 months for first‐line endocrine therapy. We further compared the predictive accuracy of optimal cut‐off values in our study and conventional cut‐off values for primary/secondary resistance from ABC3 guidelines^2^ (Figure 3). Results revealed no significant difference between the two classifications. Univariate analysis indicated patients who had longer duration of prior endocrine therapy (≥25.08 months in adjuvant setting/≥5.17 months in first‐line) reached a longer PFS when receiving fulvestrant 500 mg (Figure 4, *p* = 0.038). Multivariate analysis demonstrated that duration of prior endocrine therapy was also an independent predictive factor for PFS of fulvestrant (Table 4).

**TABLE 4:** Univariate and multivariate analysis of factors predicting PFS of fulvestrant 500 mg in patients progressed on adjuvant/first‐line endocrine therapy

	N	Event	Univariate			Multivariate		
			HR	95%CI	*p*	HR	95%CI	*p*
Age								
<65 y	122	100	0.7	0.5‐1.1	0.14			
≥65 y	37	25						
Menstrual state								
Postmenopausal	136	109	0.6	0.3‐1.1	0.061			
Premenopausal	23	16						
Liver metastasis								
No	118	94	1.3	1.0‐1.7	0.032*	1.5	1.1‐2.2	0.04*
Yes	41	31						
Lung metastasis								
No	90	73	0.9	0.6‐1.3	0.53			
Yes	69	52						
Bone alone metastasis								
No	130	103	0.67	0.4‐1.1	0.096			
Yes	29	22						
Line of endocrine therapy for MBC								
1	59	46	1.6	1.1‐2.3	0.013*	1.4	1.0‐2.2	0.027*
2	100	79						
Previous endocrine therapy								
TAM/TOR	29	22	1.6	1.0‐2.6	0.042*	1.3	0.7‐2.3	0.39
AI/Both	120	95						
Duration of last endocrine therapy								
≥25.08 m in adjuvant/≥5.17 m in first‐line setting	116	93	0.6	0.4‐0.9	0.038*	0.6	0.4‐1.0	0.04*
<25.08 m in adjuvant /<5.17 m in first‐line setting	43	32						
Previous lines of chemotherapy for MBC								
0	87	68	1.4	1.0‐2.0	0.054			
≥1	72	57						

Abbreviations: CI, confidence interval; HR, hazard ratio; MBC, metastatic breast cancer.

* *p* < 0.05 is considered significant.

### 3.3 Safety

Six patients discontinued the treatment due to adverse events (AEs). One patient experienced grade 3 anorexia and discontinued the treatment. One patient stopped the treatment due to grade 3 dizziness, grade 2 nausea, grade 1 fever, and grade 1 arthralgia. These symptoms were discontinued after treatment and were considered as AEs but not symptomatic progression. One patient stopped the treatment due to grade 3 fatigue. One patient discontinued the treatment due to grade 3 blood bilirubin increase and grade 1 AST increase. Two patients stopped the drug due to grade ≥3 anemia. All patients were recovered after discontinuation of the drug and had palliative therapy for these symptoms.

## 4 DISCUSSION

While emerging evidences confirm fulvestrant as a novel endocrine therapy due to its favorable efficacy, recent studies are focusing on its “real efficacy” in clinical practice with many other “outside trial” factors. Our study included 252 patients using fulvestrant 500 mg in real‐world medical practice, and indicated a median PFS of 5.8 months and median OS of 35.9 months. In addition, CBR was 41.3%. We also explored the potential determinants of PFS by multivariable analysis, and showed that no liver metastasis, bone alone metastasis, first‐line fulvestrant administration, and sensitive to prior endocrine therapy were significantly associated with longer PFS. The optimally predictive cut‐off values of last‐line endocrine therapy duration by survival ROC analysis were 25.08 months for adjuvant endocrine therapy and 5.17 months for first‐line endocrine therapy. This study provided insights into the practical administration of fulvestrant 500 mg and provided detailed data in Chinese patients.

Compared with previous non‐interventional studies, our study showed consistent efficacy based on large population size (Table S1). Ishida et al. retrospectively analyzed 117 patients who were treated with fulvestrant 500 mg.^13^ Results revealed that 29.1% patients had liver metastasis and 75% patients received ≥1 lines palliative chemotherapy, which was 28.9% and 56.0% in our study. It indicated that CBR was 41.9% and median time to progression (TTP) was 6.1 months, which were similar to our study (CBR 41.3%, PFS 5.8 months). Results of multivariate analysis showed that duration of first‐line endocrine therapy was a determinant of TTP. In another Italian observational study, 163 patients were enrolled who received ≤2 lines of endocrine therapy for MBC.^14^ The study demonstrated CBR of 61%, median PFS, and OS of 7 and 35 months, respectively. Visceral involvement, endocrine sensitivity, and previous endocrine therapy were considered as prognostic factors for PFS by multivariable analysis. Our study enrolled 31.0% patients who received ≥3 lines of endocrine therapy prior to fulvestrant and showed lower median PFS and CBR. Similarly, our study indicated that PFS was longer in patients who received fulvestrant 500 mg in early lines of endocrine therapy and with no liver metastasis. However, this Italian study did not show whether previous lines of chemotherapy for MBC were associated with PFS because all patients enrolled in this study only received at most 1 line of chemotherapy. Kawaguchi et al. analyzed 1072 patients who received fulvestrant 500 mg from 16 registries in Japan.^15^ This study showed a median time to treatment failure (TTF) of 5.4 months. Earlier fulvestrant 500 mg use, longer period from MBC diagnosis to fulvestrant 500 mg, and no prior palliative chemotherapy for MBC were associated with significantly longer TTF. Overall, these studies, including our study, suggest that liver metastasis, bone alone metastasis, lines of endocrine therapy for MBC, lines of palliative chemotherapy, previous endocrine therapy, and endocrine sensitivity for MBC were determinants for PFS/TTP. Discrepancy in these factors may result from differences in sample size, patient characteristics and variables included in the multivariable analysis.

Fulvestrant 500 mg showed a favorable efficacy in patients with no liver metastasis. FALCON study^6^ demonstrated a significantly longer PFS in patients without visceral disease compared with those with visceral disease (HR 0·59, 95% CI 0·42–0·84, mPFS for fulvestrant group 22·3 months and for anastrozole group 13·8 months). The influence of liver metastasis on PFS was also identified in CONFIRM study.^7^ Patients with no visceral involvement benefited more from fulvestrant 500 mg compared with fulvestrant 250 mg. These data suggest that visceral metastasis can be a predictor of PFS in fulvestrant 500 mg therapy. Our study pointed out that liver metastasis could be a good determinant of PFS in patients treated with fulvestrant 500 mg. In this study, patients with no liver metastasis showed a significantly longer PFS than those with liver metastasis (mPFS 7.1 vs. 3.7 m, *p* = 0.000), and was further confirmed by multivariable analysis (Table 3). Liver metastasis showed highest frequency of visceral metastasis and approximately 50% patients may develop liver metastasis.^16^ Patients who develop visceral metastases to the liver generally have poorer outcomes than patients with metastases to bone or even lung, with a median survival <6 months.^17^, ^18^, ^19^ Patients with liver metastasis represent a high‐risk group of MBC patients with poor prognosis and may not respond well to conventional endocrine therapy.^6^, ^7^, ^20^ Thus, liver metastasis is not only a prognostic factor, but also a favorable factor for predicting the efficacy of fulvestrant 500 mg in MBC patients.

Earlier fulvestrant 500 mg use, especially first‐line therapy, demonstrated an enhanced benefit and prolonged PFS by 7 months (11.9 vs. 4.7 months, *p* = 0.000). Though there were no comparisons in prospective clinical trials between different lines of fulvestrant 500 mg, median PFS was 6.5 months in second‐line patients from CONFIRM study^7^ and 16.6 months in first‐line patients from FALCON study,^6^ demonstrating a numerically longer PFS in early fulvestrant usage. In addition, several retrospective studies showed that previous endocrine therapy or earlier fulvestrant 500 mg use was significantly associated with PFS of fulvestrant 500 mg.^14^, ^15^ Subsequent lines of endocrine therapy increased the acquired resistance. Therefore, first‐line administration of fulvestrant 500 mg is suggested in the treatment MBC.

In our study, median PFS was decreased significantly with successive chemotherapy regimen. The results were similar to the previous studies. FALCON study^6^ showed that fulvestrant 500 mg achieved a favorable efficacy in patients with no previous chemotherapy for MBC compared with anastrozole. Furthermore, Kawaguchi et al.^15^ and Araki et al.^20^ confirmed a significant association between PFS of fulvestrant 500 mg and previous palliative chemotherapy. These results suggested that fulvestrant 500 mg given before palliative chemotherapy could reach a longer PFS for patients with low tumor burden and /or no need for a quick response. However, we noted that patients who received fulvestrant 500 mg after one line chemotherapy for MBC achieved a median PFS of 5.8 months, which reached the median PFS of overall population, indicating that fulvestrant 500 mg has substantial activity as maintenance therapy subsequent to chemotherapy. FANCY study,^21^ a single‐arm phase 2 trial, explored fulvestrant 500 mg as maintenance endocrine treatment after chemotherapy. Results revealed that 58 patients with disease control after first‐line chemotherapy reached a median PFS of 16·1 months (since fulvestrant treatment) and a CBR of 76%. Though this study was limited to its sample size and power of phase 2 trial, it still proved that fulvestrant 500 mg was active as maintenance therapy in non‐progressive patients with HR‐positive MBC after first‐line chemotherapy. Our study did not estimate the median PFS of fulvestrant 500 mg after first‐line chemotherapy, but only analyzed the patients who received one previous chemotherapy regimens (no limitation to lines of previous endocrine therapy), which showed a median PFS of 5.8 months. Nevertheless, all these results suggested that fulvestrant 500 mg is recommended as maintenance endocrine therapy after first‐line chemotherapy.

On one hand, multivariable analysis showed no significant association of menstrual state with PFS. These results demonstrated that postmenopausal and premenopausal HR+MBC patients showed similar duration of disease control when treated with fulvestrant 500 mg. Previous phase 2‐3 randomized clinical trials enrolled only postmenopausal patients and there were only few studies on the efficacy of fulvestrant 500 mg in premenopausal patients. As the mean age for diagnosing breast cancer in China is 45–55 years, which is considerably younger than the western women,^22^ the treatment of premenopausal breast cancer patients remains to be a tough task in China. Recently, PROOF study, is enrolling premenopausal advanced breast cancer patients and compares efficacy of goserelin plus fulvestrant 500 mg with goserelin plus anastrozole as first‐line endocrine therapy (NCT02072512). This study will provide more data regarding fulvestrant 500 mg in the premenopausal advanced breast cancer patients.

On the other hand, patients with different previous endocrine therapies, whether TAM/TOR, AI or both, showed no statistically significant difference in PFS. CONFIRM study^7^ showed that patients who had prior TAM/TOR therapy favored the use of fulvestrant 500 mg compared with fulvestrant 250 mg, but this trend was not observed in patients who had prior AI. In China, CONFIRM study^23^ demonstrated that patients who had previous endocrine therapy were consistent with the overall effect on PFS. A numerically longer PFS was observed in the TAM/TOR group than in the AI group in patients treated with fulvestrant 500 mg (8.1 vs. 5.8 months, respectively). We demonstrated a similar efficacy in the post‐TAM/TOR and post‐AI patients. Numerical differences may result from different ages and high relapse risks in patients who chose AI rather than TAM/TOR agents as adjuvant endocrine therapy.

Using survival ROC analysis, the optimal cut‐off values were shown as 25.08 months for adjuvant endocrine therapy and 5.17 months for first‐line endocrine therapy. The optimal cut‐off values showed a numerically different AUC compared with the conventional cut‐off values used in the ABC3 guideline and previous clinical trials. Survival ROC is a method for displaying sensitivity/specificity and optimal cut‐off point of a continuous diagnostic marker for time‐dependent disease outcomes. This demonstrated that the ABC clinical definition of primary and secondary endocrine resistance corresponded with the predicted optimal cut‐off point by survival ROC. Primary and secondary endocrine resistance can separate the patients who will benefit from fulvestrant 500 mg or not, which is helpful in guiding selection of endocrine treatment only or with targeted agents. However, no validation set is available for further identification in this study.

Though the combination with CDK4/6 inhibitor extended the PFS of ER+MBC patients,^8^, ^9^ overall survival was not improved in entire trial patients except those who were sensitive to previous endocrine therapy, postmenopausal, ≥65 years old, and had a DFI > 24 months.^24^ For patients who will not gain a survival benefit from CDK4/6 inhibitor, fulvestrant alone may be an alternative considering a big burden for patients to take up a long course of CDK4/6 inhibitor treatment. Previous study showed that adding palbociclib to letrozole was estimated to cost an additional CHF342,440 and gain 1.14 quality‐adjusted life years, resulting in an incremental cost‐effectiveness ratio (ICER) of CHF301,227/QALY gained.^25^ As CDK4/6 inhibitor is not covered in the national medical insurance in China, the addition of CDK4/6 inhibitor may cause a discounted cost‐effectiveness. Therefore, fulvestrant alone will still fit for some selected patients who are not available to or will not benefit from CDK4/6 inhibitor.

Our study has a few limitations. First, our study is a retrospective research and bias is inevitable due to its nature. Second, we enrolled patients who were treated with only fulvestrant 500 mg but did not include any control group. Third, our patients were from single‐center, which would lead to selection bias and confounding factors.

In brief, this study was a pioneer work, and provided first‐hand data regarding the efficacy and potential determinants of fulvestrant 500 mg in Chinese patients with HR+MBC. No liver metastasis, first‐line fulvestrant administration, and sensitive status to last endocrine therapy prior to fulvestrant were significantly associated with longer PFS. Cost of fulvestrant 500 mg may be a major factor for poor compliance. The study showed better cut‐off values for last endocrine therapy duration was 25.08 months for adjuvant endocrine therapy and 5.17 months for first‐line endocrine therapy, but further studies are needed to confirm this. Yet, the results should be interpreted considering the nature of retrospective study. We expect more studies to confirm the predictive factors and biomarkers for better use of fulvestrant 500 mg.

In conclusion, fulvestrant 500 mg is effective and has excellent safety profile in HR+MBC patients. The duration of prior endocrine therapy is a predictive factor of fulvestrant and the optimal cut‐off values better guide the selection of endocrine therapy. No liver metastasis, bone alone metastasis, and first‐line of fulvestrant also indicate better efficacy of fulvestrant.

## DECLARATIONS

The Ethics Committee and Institutional Review Board of Fudan University Shanghai Cancer Center approved this study. The need for written informed consent was waived as it is a retrospective study.

## CONFLICT OF INTERESTS

The authors declare that they have no competing interests.

## AUTHOR CONTRIBUTIONS

Biyun Wang and Xichun Hu: study conception and design; Jian Zhang, Jun Cao, Leiping Wang, Biyun Wang, and Xichun Hu: provision of study material or patients; Yannan Zhao, Yi Li, Chengcheng Gong, and Xieyi Zhao: Collection and/or assembly of data. Yannan Zhao and Yi Li: Data analysis, interpretation, and manuscript writing; All authors have contributed to research platform design and management and approved the final manuscript. This research is registered at clinicaltrials.gov (NCT 03708432).

## Supporting information

Table S1

## Data Availability

The datasets generated and analyzed during the current study are not publicly available due to hospital policy but are available from the corresponding author on reasonable request.
