# Supplementary material for: Optimal duration of prior endocrine therapy predicts the efficacy of Fulvestrant in a real‐world study for patients with hormone receptor‐positive and HER2‐negative advanced breast cancer
Source: Cancer Med. 2020 Oct 6;9(23):8821–31. doi: 10.1002/cam4.3491 (PMC7724295; doi:10.1002/cam4.3491)
Supplement: Supplementary file 1 — Table S1 [file CAM4-9-8821-s001.docx]

**Table S1. Real-world studies of fulvestrant 500mg for metastatic breast cancer**

| **Study** | **Study setting** | **Number of patients** | **Clinical benefit rate (%)** | **Median TTP/PFS**  **(months)** | **Median OS**  **(months)** |
| --- | --- | --- | --- | --- | --- |
| Our study | All lines | 252 | 41.3 | 5.8 | 35.9 |
| Ishida et al.^13^ | All lines | 117 | 41.9 | 6.1 | Unknown |
| Moscetti et al.^14^ | ≤2 lines | 163 | 61 | 7 | 35 |
| Kawaguchi et al.^15^ | All lines | 1072 | Unknown | 5.4 | Unknown |
| Araki et al.^20^ | All lines | 194 | Unknown | 5.48 | Unknown |
